# Global Diversification and Distribution of Coronaviruses With Furin Cleavage Sites

**DOI:** 10.3389/fmicb.2021.649314

**Published:** 2021-10-07

**Authors:** Xiaotong Liu, Qunfu Wu, Zhigang Zhang

**Affiliations:** State Key Laboratory for Conservation and Utilization of Bio-Resources in Yunnan, School of Life Sciences, Yunnan University, Kunming, China

**Keywords:** COVID-19, SARS-CoV-2, furin cleavage site, coronaviruses, diversification

## Abstract

Knowledge about coronaviruses (CoVs) with furin cleavage sites is extremely limited, although these sites mediate the hydrolysis of glycoproteins in plasma membranes required for MERS-CoV or SARS-CoV-2 to enter cells and infect humans. Thus, we have examined the global epidemiology and evolutionary history of SARS-CoV-2 and 248 other CoVs with 86 diversified furin cleavage sites that have been detected in 24 animal hosts in 28 countries since 1954. Besides MERS-CoV and SARS-CoV-2, two of five other CoVs known to infect humans (HCoV-OC43 and HCoV-HKU1) also have furin cleavage sites. In addition, human enteric coronavirus (HECV-4408) has a furin cleavage site and has been detected in humans (first in Germany in 1988), probably *via* spillover events from bovine sources. In conclusion, the presence of furin cleavage sites might explain the polytropic nature of SARS-CoV-2- and SARS-CoV-2-like CoVs, which would be helpful for ending the COVID-19 pandemic and preventing outbreaks of novel CoVs.

The ongoing epidemic of coronavirus disease 2019 (COVID-19), caused by severe acute respiratory syndrome coronavirus 2 (SARS-CoV-2) began in late 2019. Since then, it has spread all over the world at an alarming rate, causing more than 199 million infections and 4 million deaths by August 2021 (WHO, 2021). The severity of the epidemic poses huge threats to the global economy and health.

Before the outbreak, six types of coronaviruses were known to infect humans: four low-pathogenicity coronaviruses (HCoV-229E, HCoV-NL63, HCoV-OC43, and HCoV-HKU1) and two highly pathogenic zoonotic pathogens (MERS-CoV and SARS-CoV), all of which are betacoronaviruses except HCoV-229E and HCoV-NL63 (two alphacoronaviruses) (Zhu et al., 2020). During the last two decades, two of these human coronaviruses (HCoVs), SARS-CoV and MERS-CoV, extensively infected humans, causing major outbreaks of respiratory disease with high mortality rates. The novel coronavirus SARS-CoV-2, another betacoronavirus, is the seventh type known to infect humans and the third type that is highly pathogenic (Noy-Porat et al., 2020).

The entry of coronavirus into a host cell mainly depends on binding of the viral spike (S) proteins to the receptor, angiotensin-converting enzyme 2 (ACE2) (Lan et al., 2020; Shang et al., 2020). The S protein is a highly glycosylated type I transmembrane protein, with an N-terminal S1 subunit responsible for binding the receptor and a C-terminal S2 subunit that mediates fusion of the viral and cell membranes (Belouzard et al., 2012; Saputri et al., 2020). At the junction of S1 and S2, the specific sequence motif PRRAR can be recognized and cleaved by host furin protease.

Millet and Whittaker (2014) confirmed that MERS-CoV carries a furin cleavage site and found that overexpression of furin protease and the viral receptor DPP4 in low-susceptibility HEK-293T cells markedly increased infection rates (Millet and Whittaker, 2014). They also found that infection can be enhanced by an unusual two-step, fusion-mediating furin activation process, suggesting that hydrolysis of glycoproteins on the cell membrane mediated by the furin-like cleavage site is important for the virus to enter the cell.

Like MERS-CoV (but not SARS-CoV), SARS-CoV-2 was found to carry a furin cleavage site (NSPRRAR↓SV) because of a 12-nucleotide insertion (Coutard et al., 2020; Walls et al., 2020; Zhang et al., 2020), which has led to speculation that this cleavage site may be artificial. However, analysis of coronavirus sequences has shown that natural evolution of the furin cleavage site in the SARS-CoV-2 S protein is highly probable (Wu and Zhao, 2020). The furin-cleavage motif of SARS-CoV-2 S protein is recognized and cleaved during virus packaging, which can promote viral infectivity as a result of the conformational change required for exposure of the protein’s receptor-binding domain (Wrobel et al., 2020; Peacock et al., 2021). Another study has suggested that co-expression of neuropilin-1 (NRP1), which is known to bind furin-cleaved substrates, with ACE2 and TMPRSS2, can also enhance SARS-CoV-2 infectivity (Daly et al., 2020).

Evidence obtained from both *in vivo* and *in vitro* studies has confirmed that the furin cleavage site plays key roles in SARS-CoV-2’s infectivity and pathogenicity. Several studies have shown that the site promotes the S protein’s mediation of virus–cell fusion (Cheng et al., 2020; Hoffmann et al., 2020; Xia et al., 2020). A mutant that lacks the PRRA motif reportedly has higher replication rates than wild-type in Vero E6 cells, but lower rates in a human respiratory cell line (Johnson et al., 2021). *In vivo* experiments indicate that loss of the furin cleavage site in the S protein considerably attenuates the pathogenesis and infectivity of SARS-CoV-2, by reducing its replication rates in early stages of infection in both hamsters and K18-hACE2 transgenic mice, which express human ACE2 (Johnson et al., 2021). Moreover, Peacock et al. (2021) found that mutant SARS-CoV-2 with a deleted furin cleavage site had low replicability in the upper respiratory tracts of model animals (ferrets) and was not transmitted to cohoused sentinel animals (Peacock et al., 2021). Thus, presence of a furin cleavage site in S protein is crucial for replication (Papa et al., 2021), pathogenesis, and transmission of SARS-CoV-2 (Johnson et al., 2020). This might explain why SARS-CoV-2 has higher transmissibility than SARS-CoV.

Possible host ranges and pathogenicities of some CoVs with furin cleavage sites have also been assessed (Millet and Whittaker, 2015), but knowledge of the spatial–temporal distribution and evolutionary history of CoVs with these sites is still extremely limited. Clearly, furin inhibitors could be potent therapeutic agents for treating SARS-COV-2 infection (Cheng et al., 2020), so it is crucial to obtain detailed knowledge of CoVs with furin cleavage sites to end the COVID-19 pandemic and prevent new potential outbreaks of pathogenic CoVs.

To assist such efforts, we retrieved S protein sequences of 2,297 coronaviruses of four genera (*Alphacoronavirus*, *Betacoronavirus*, *Gammacoronavirus*, and *Deltacoronavirus*) from the National Genomics Data Center (National Genomics Data Center Members and Partners, 2020; see Supplementary Materials). We removed sequences lacking furin cleavage sites and sequences with furin prediction scores below 0.5 using ProP (v1.0) software (Duckert et al., 2004). We then investigated the evolutionary history of the remaining 249 CoVs with diversified furin cleavage sites, and analyzed both their known host ranges and geographical distributions since 1954 (Figures 1, 2, Supplementary Figure 1, and Supplementary Table 1). The findings provide valuable insights for formulating global plans and preventing outbreaks of novel CoVs that may endanger both human beings and animals. It should be noted that the Malayan horseshoe bat (*Rhinolophus malayanus*) hosts a recently discovered bat CoV (RmYN02) with a putative furin cleavage site that is reportedly the most closely related to SARS-CoV-2 (Zhou H. et al., 2020). However, it was assigned a poor furin score of 0.111 in this study and thus excluded from further analysis. Based on sequence alignments, we also excluded Delta-CoVs from the dataset as their predicted furin cleavage sites are not at the spike S1/S2 location recorded in members of the Alpha, Beta, and Gamma genera. We found that 1 SARS-CoV-2 representative (Genbank Accession No. MN908947) and 248 other CoVs carry 86 high-confident furin cleavage sites (prediction score: 0.89 ± 0.12, mean ± SD), which are members of the Alpha-CoV, Beta-CoV, or Gamma-CoV genera and have been detected in *Homo sapiens* and 23 other animal hosts (Figure 1).

**FIGURE 1:**
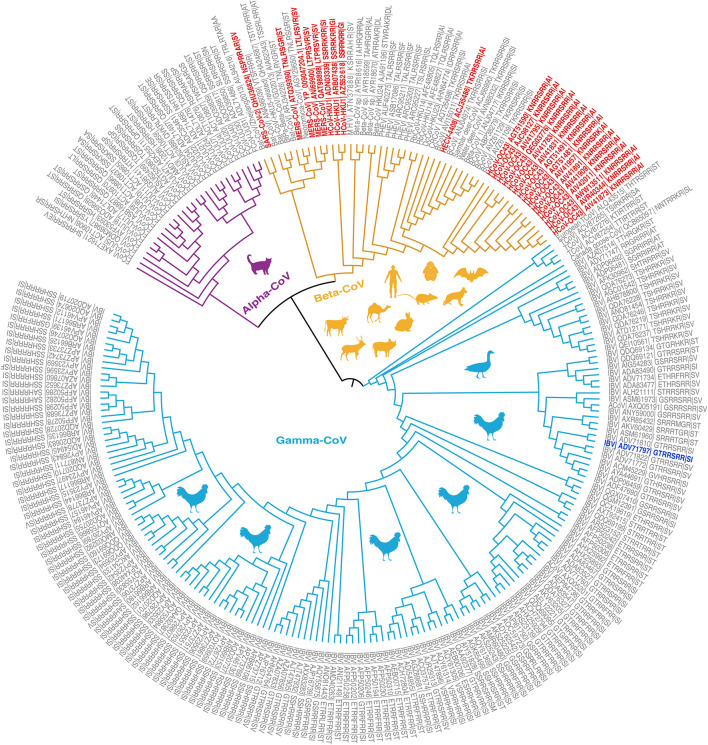
Phylogenetic relationships of coronavirus spike proteins with furin cleavage sites. For information regarding the proteins (see Supplementary Tables 1, 2). The branches are colored according to virus genus (purple = Alpha-CoV, yellow = Beta-CoV, light blue = Gamma-CoV). Animal shapes indicate the viruses’ main hosts and are colored according to the genus of virus they host. The leaves show the virus species, GenBank Accession numbers, and furin cleavage site motifs. Four coronaviruses with furin cleavage sites known to infect humans (HCoV-OC43, HCoV-HKU1, MERS-CoV, and SARS-CoV) and a human enteric coronavirus 4408 (HECV-4408) first detected in Germany in 1988 are marked in red. The first recorded coronavirus with a furin cleavage site was detected in *Gallus* and is marked in blue.

**FIGURE 2:**
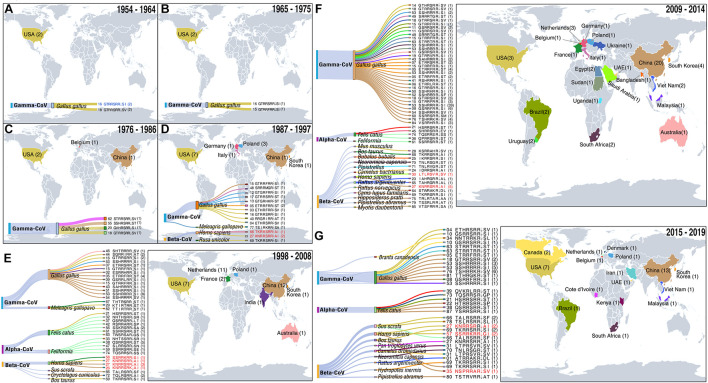
Temporal–spatial distribution of CoVs with furin cleavage sites. For information on the sites (see Supplementary Table 1). **(A–G)** Sources of coronaviruses recorded in indicated periods with furin cleavage sites displayed in Sankey plots. Level 1 shows the virus genera, level 2 shows animal hosts of CoVs, and colors of the lines correspond to colors on the map of the countries in which the CoVs with indicated furin cleavage sites were detected. After level 2, the furin cleavage site numbers and motifs are shown. The numbers in parentheses refer to frequencies of furin cleavage motifs. Numbers on the map show numbers of types furin cleavage sites detected in the indicated countries. Furin cleavage sites from four CoVs known to infect humans (HCoV-OC43, HCoV-HKU1, MERS-CoV, and SARS-CoV) and human enteric coronavirus 4408 (HECV-4408), first detected in Germany in 1988, are marked in red. The first coronavirus with a furin cleavage site, which appeared in *Gallus*, is marked in blue. Records of coronaviruses with furin cleavage sites from 1954 to 2008 including five time periods (11 years per period) **(A–E)**, 2009–2014 **(F)** and 2015–2019 **(G)**.

Our results indicate that the first CoV with a furin cleavage site was detected in 1954 (Figure 2A and Supplementary Table 1) and provide a detailed history spanning more than 60 years of records of CoVs with these sites, in decadal steps as described below and illustrated in Figure 2.

## 1954–1964

CoVs with furin cleavage sites emerged at least 66 years ago. The first to be recorded was an infectious bronchitis virus (IBV: Genbank Accession No. ADV71797) with a GTRRSRR↓SI furin cleavage site. This was a Gamma-CoV, isolated from *Gallus gallus* in the United States in 1954 (Figure 2A). Genomic information on this IBV, which can infect domestic chickens’ respiratory tracts, was published in 2011 (Thor et al., 2011). During the decade 1954–1964, two types of furin cleavage sites in IBV infecting *G. gallus* were recorded (GTRRSRR↓SI and GTRRSRR↓SV), both of which reportedly arose in North America (Figure 2A).

## 1965–1975

During the period 1965–1975 (Figure 2B), IBV was still detected in *G. gallus* in the United States. In addition, a new type of furin cleavage site (GTRRFRR↓SI) was detected in an IBV (Genbank Accession No. ADA83547) isolated in 1972. Its whole genome was published nearly 40 years later by McKinley et al. (2011), who mentioned that high mutation and replication rates may contribute to rapid generation of new variants of the virus (McKinley et al., 2011). Relationships of the 86 identified high-confidence furin cleavage sites also indicate that shifts and replications have occurred in these sites of all three coronavirus genera (*Alphacoronavirus*, *Betacoronavirus*, and *Gammacoronavirus*) (Supplementary Figure 1). This confirms the sites’ natural occurrence, and the possibility that new types of furin cleavage sites arose spontaneously in the United States.

## 1976–1986

From 1976 to 1986 (Figure 2C), *G. gallus* infections by IBV were successively recorded in North America, Europe, and Asia. Three new types appeared: one in the United States, one in Belgium, and one in China. The furin cleavage site GTRRSRR↓SV detected in 1964 was detected again in 1981, in the same host but a different virus strain (Genbank Accession No. ADP06459) in the United States. In addition, a new type of furin cleavage site (GVHRSRR↓SI) was found in an IBV (Genbank Accession No. ACM45229) in the United States in 1976. The occurrence of respiratory IBVs with furin cleavage sites had been confirmed in the United States in the 1950s (Reddy et al., 2015), and other variants subsequently emerged. Nephropathogenic infectious bronchitis virus (NIBV) strain B1648 with a furin cleavage site (STRRSRR↓SV) was first isolated in 1984 in Belgium and its full-length genome, obtained by next-generation sequencing (NGS), was published in 2015 (Reddy et al., 2015). In 1985, a new type of furin cleavage site (SSHRSRR↓ST) was discovered in Guangxi province, China (Genbank Accession No. AGK85499) but no related study was published. This was the earliest recorded coronavirus with a furin cleavage site detected in China.

## 1987–1997

During the decade from 1987 to 1997, the geographical distribution of CoVs with furin cleavage sites began to expand strongly (Figure 2D). Six new types of these sites were identified in the United States, five in Europe (one in Germany, one in Italy, and three in Poland), and two in Asia (one in China and the other in South Korea). Notably, the human enteric coronavirus 4408 (HECV-4408, Beta-CoV) with a furin cleavage site (TKRRSRR↓AI) was isolated from diarrhea fluid of a child in Germany in 1988 (Bradburne, 1969; Zhang et al., 1994; Figures 1, 2). HCoV-B814 was the first coronavirus to be isolated from humans (in nasal discharges) by Tyrrell and Bynoe (1965), and a similar virus (human coronavirus 229E, HCoV-229E) was isolated shortly thereafter by Hamre and Procknow (1966) in the United States (Hamre and Procknow, 1966). HCoV-229E was the first coronavirus confirmed to infect humans, but it does not contain a furin cleavage site. Another furin cleavage site (KNRRSRR↓AI) appeared in HCoV-OC43 (Beta-CoV), which was first discovered in the nasopharyngeal wash of a common cold patient in the United States in 1967 (McIntosh et al., 1967; Lau et al., 2011; Su et al., 2016), and recurred in the United States in 1991 and 1996. Subsequent studies revealed that HCoV-OC43 may have originated in rodents, with subsequent transmission to humans through cattle as intermediate hosts (Lau et al., 2015; Ye et al., 2020). Nucleotide sequence analysis revealed more than 99% nucleotide and amino acid homologies between spike proteins of HECV-4408 and bovine coronavirus (BCoV), indicating that HECV-4408 is more closely related, antigenic ally and genetically, to BCoV than to HCoV-OC43 (Figure 1; Zhang et al., 1994). Moreover, another Beta-CoV (Samba deer coronavirus) with a furin cleavage site was isolated from samba deer (*Ruse unicolor*) and may have been causally associated with outbreaks of winter dysentery and sporadic diarrhea in ruminants in the United States in 1993 and 1994 (Alekseev et al., 2008). In addition, 11 types of furin cleavage sites were detected in Gamma-CoVs, including nine new types detected in bird hosts (10 in *G. gallus* and one in *Meleagris gallopavo*) in five countries (China, South Korea, Italy, Poland, and the United States). In a few cases, Beta-CoVs with these sites were also found in mammals, including *Homo sapiens* (the United States and Germany) and *R. unicolor* (the United States).

## 1998–2008

During the decade from 1998 to 2008, CoVs containing furin cleavage sites were surveyed worldwide in diverse animal hosts (Figure 2E). Gamma-CoVs with furin cleavage sites (distributed in Australia, China, India, South Korea, France, Poland, and the United States) were still limited to avians and showed high diversity. In addition, a feline coronavirus (FCoV, Alpha-CoV) with a furin cleavage site (HSRRSRR↓ST) was found in the United States in 1998, and 16 containing 11 types of furin cleavage sites were recovered from *Felis catus* in The Netherlands in 2007 and 2008. These findings clearly indicate that FCoVs have high host specificity, and some strains can cause highly fatal feline infectious peritonitis (Taharaguchi et al., 2012). Furin cleavage sites derived from Beta-CoVs were also detected in mammals, including *H. sapiens*, *Oryctolagus cuniculus*, *Sus scrofa*, and *Bos taurus*. Numbers of recorded cases of Beta-CoVs infecting herbivores began to increase during this period. Moreover, a new coronavirus named HCoV-HKU1 (Genbank Accession No. ADN03339) with a furin cleavage site (SSRRKRR↓SI) that can infect humans was first discovered in 2004 in China (Woo et al., 2005) and subsequently found in France in 2005 (Figure 1). HCoV-HKU1 can cause an upper respiratory disease with symptoms of the common cold, potentially advancing to pneumonia and bronchiolitis (Lim et al., 2016). Phylogenetic analysis showed that HCoV-HKU1 likely originated from rodents (Lau et al., 2015; Ye et al., 2020). During this decade, the major SARS-CoV outbreak may have driven large-scale detection of CoVs.

## 2009–2014

During this 5-year period (Figure 2F), Gamma-CoV with furin cleavage sites were detected in 13 countries located in many parts of the world (Australia, Brazil, China, Egypt, Italy, Malaysia, Poland, South Africa, South Korea, Sudan, the United States, Ukraine, and Uruguay). These sites had extremely high diversity (up to 21 variants, including 13 that had not been previously identified). The furin cleavage sites in Alpha-CoVs included a motif (HSRRSRR↓ST) found in 1998 (Genbank Accession No. ACT10959) in the United States, which was detected again in Belgium in 2013, and two novel motifs (Genbank Accession Nos. AEK25511 and AXE71621). Beta-CoVs with furin cleavage sites were detected in diverse mammalian species (*Bubalus bubalis*, *H. sapiens*, *Rattus argentiventer, R. norvegicus*, *Canis lupus familiaris*, *B. taurus*, *Pipistrellus*, *Camelus bactrianus*, *Mus musculus*, and four bat species). Bats were thought to be natural reservoirs of the SARS-like CoVs (Li et al., 2005) and four with furin cleavage sites were found in four bat species (*Myotis daubentoniid*, *Hipposideros pratti*, *Pipistrellus abramus* in China, and *Neoromicia capensis* in South Africa). MERS-CoV, a zoonotic Beta-CoV that originated in bat (*Taphozous perforates*), can infect humans and camels (Memish et al., 2013; Wong et al., 2019). MERS-CoV was first identified in Saudi Arabia in 2012 (Zaki et al., 2012), following the death of a patient due to a severe respiratory illness. MERS-CoV cases in Saudi Arabia were reported for the first time in September 2012 (Zaki et al., 2012). Camels (*C. bactrianus*) are considered to be intermediate hosts of MERS-CoV (Perera et al., 2013; Azhar et al., 2014; Mostafa et al., 2020). In contrast to the common-cold coronaviruses known as HCoV-OC43 and HCoV-HKU1, MERS-CoV can infect humans on a massive scale with high fatality. Millet and Whittaker (2014) clearly showed that inhibition of furin activity can decrease MERS-CoV S-mediated entry and infection, providing the first confirmation of the furin cleavage site’s role in disease caused by the virus (Millet and Whittaker, 2014). In summary, CoVs with new furin cleavage sites were detected in many countries, more furin cleavage sites were recorded in avian coronaviruses, and Beta-CoVs with furin cleavage sites were shown to have diverse hosts (mainly mammals). In addition, strongly pathogenic Beta-CoVs with furin cleavage sites began to appear, giving the first strong warnings that they could pose potent threats to humans.

## 2015–2019

In the 5 years from 2015 to 2019 (Figure 2G), Gamma-CoVs with furin cleavage sites were still mostly found in *G. gallus*, but one case was recorded in *Branta canadensis* in 2017 in Canada (Papineau et al., 2019). This indicated that wild birds might be threatened by CoVs. Infections of *Felis catus* by Alpha-CoVs with four types of furin cleavage sites continued to occur in Brazil (2015), Belgium (2015), Denmark (2015), and China (2016 and 2018). In addition, 11 Beta-CoVs with furin cleavage sites, including seven novel types, were recorded. MERS-CoVs with furin cleavage sites were detected from *Neoromicia capensis* in South Africa and *Camelus dromedarius* in both Kenya and the United Arab Emirates. Moreover, a virus named “Neo-CoV” with a furin cleavage site (Genbank Accession No. AGY29650), which is very closely related to MERS-CoVs, was isolated from Cape serotine (*Neoromicia capensis*) in South Africa in 2011 (Figure 1), and Patrono et al. (2018) found that HCoV-OC43 can infect *Pan troglodytes verus* (Patrono et al., 2018). HCoV-OC43, which infected humans in 2017 and 2019 in the United States, was also detected in *Pan troglodytes verus* in Cote d’Ivoire in 2017, suggesting that it may be an intermediate host of HCoV-OC43. HCoV-HKU1 occurred in 2016–2017 in the United States, and HKU5-related bat coronavirus was detected in *Pipistrellus abramus* in China. This indicates that bats may be natural sources of HCoV-HKU1-like CoVs. Other Beta-CoVs with furin cleavage sites were found in *S. scrofa* in the United States and the Netherlands, *B. taurus* (United States and China), *R. argentiventer* (Vietnam), and *Hydropotes inermis* (South Korea). In 2019, SARS-CoV-2 [which has a NSPRRAR↓SV furin cleavage site, and bat or pangolin are suspected as natural hosts (Zhang et al., 2020; Zhou P. et al., 2020)] caused the COVID-19 pandemic and sent stronger warnings for the threat of CoVs with furin cleavage sites to human beings or animals.

## Conclusion

Thorough structural understanding of SARS-CoV-2 is crucial to control the global outbreak of the virus and prevent outbreaks of related viruses. However, the furin cleavage site’s role did not receive sustained attention following the discovery of coronaviruses until the COVID-19 outbreak in 2019. Our results show that 86 types of furin cleavage sites have been detected in strains of three coronavirus genera detected in 24 animal hosts in 28 countries since 1954, including at least 25 types in Beta-CoVs recorded in the years 1988–2019 in 14 countries (Vietnam, Bangladesh, China, South Korea, Saudi Arabia, the United Arab Emirates, Cote d’Ivoire, Uganda, Kenya, South Africa, France, Germany, the Netherlands, and United States). Most of them could cause unexpected threats to human beings or other mammals. Four of seven CoVs known to infect humans carry furin cleavage sites, including two with low pathogenicity (HCoV-OC43 and HCoV-HKU1) and two highly pathogenic zoonotic viruses (MERS-CoV and SARS-CoV-2). Moreover, evidence of frequent interchange of furin cleavage site motifs among the three coronavirus genera indicates that frequencies of recombination of CoVs’ furin cleavage sites may have been underestimated (Supplementary Figure 1). The presence of furin cleavage sites associated with changes in pathogenicity might also explain the polytropic nature of SARS-CoV-2 and SARS-CoV-2-like CoVs.

## Perspectives

The last outbreak of a human coronaviruses with a furin cleavage site before the current pandemic was the MERS-CoV outbreak in 2014. At the end of January 2020, over 2,500 laboratory-confirmed cases of MERS with more than 800 deaths (case-fatality rate: 34.3%) were reported worldwide (WHO, 2020). The reported cases of MERS were mainly in Saudi Arabia, and the outbreak did not attract global attention due to its small spread compared with the current COVID-19 pandemic. The latter poses massive global challenges, numbers of deaths due to the novel virus are still increasing, and the end of the pandemic is still unpredictable.

The origin of the novel coronavirus has also been strongly debated, and tracing SARS-CoV-2’s source is important for controlling its spread. In early stages of the outbreak, some people argued that the novel coronavirus with a furin cleavage site was an artificial virus. However, previous findings (Wu and Zhao, 2020) and our results show that CoVs with furin cleavage sites have existed since at least 1954. The global host ranges and geographical distributions of these viruses and their history of at least 60 years show that diversified furin cleavage sites do not have synthetic origins and might provide CoVs multiple pathways to infect human beings or other animals. Besides transmission from animals to humans, SARS-CoV-2 can also spread through cold food supply chains (Zhou and Shi, 2021).

Recently, genetic variants of SARS-CoV-2 have been emerging worldwide. Up to June 2021, several variants of concern for which there is evidence of an increase in transmissibility and reduced effectiveness of treatments or vaccines have been reported. These include B.1.1.7 (alpha variant), B.1.351 (beta variant), P.1 (gamma variant), and B.1.617.2 (delta variant), first identified in the United Kingdom, South Africa, Japan/Brazil, and India, respectively (CDC, 2021; Saito et al., 2021). Mutations of concern include the P681R mutation in the S protein of B.1.617.2, close to the furin cleavage site, which may increase the rate of S1/S2 cleavage and enhance viral fusion (Cherian et al., 2021; Saito et al., 2021). Fortunately, a reverse genetic system for SARS-CoV-2 has been developed to generate mutants of the virus, which could be used to examine effects of the furin cleavage site’s deletion on virus replication and facilitate analyses of the replication and pathogenicity of the virus (Xie et al., 2020; Johnson et al., 2021).

The evolution of multiple CoVs with furin cleavage sites during the last 60 years clearly highlights the need to understand roles of the site and other functional elements of SARS-CoV-2 in order to identify therapeutic targets and facilitate vaccine development. Global collaborative efforts are needed to meet these goals and help efforts to prevent further spread of SARS-CoV-2 and improve therapeutic interventions. To aid such efforts, we make the following suggestions.

1. Vaccination is the first option to counter the COVID-19 pandemic. Evidence indicated that vaccines can reduce the risk of household transmission by 40–50% (Harris et al., 2021). Vaccine development is accelerating all over the world, but there are urgent needs for more rapid production of effective COVID-19 vaccines and therapeutic agents. Moreover, even a highly effective vaccination program may not be sufficient to end the COVID-19 epidemic. People must remain vigilant and equitable distribution of vaccines around the world is crucial.
2. It is important to identify potential intermediate hosts. Intermediate hosts of SARS-CoV and MERS-CoV are palm civet (*Paradoxurus hermaphroditus*) and camels (*C. dromedarius*), respectively, but the intermediate host of SARS-CoV-2 is still unknown. The virus can reportedly be transmitted from animals to humans, so discovery of potential intermediate hosts is essential for cutting the transmission between animals and humans *via* timely and effective intervention. In addition, transmission *via* cold food supply chains cannot be neglected as SARS-CoV-2 can survive on surfaces of cold food packages up to 3 weeks (Zhou and Shi, 2021). So, countries should take urgent measures to control the spread of SARS-CoV-2 through supply chains. It also helps to prevent this pandemic from further deteriorating and could decrease loss of life and property.
3. Genetic recombination of both DNA and RNA viruses is a common phenomenon. The ability of SARS-COV-2- and SARS-COV-2-like CoVs to mutate may have been vastly underestimated, and mutations affect strains’ lethality. Thus, there are urgent needs to comprehensively clarify the pathogenic mechanism of SARS-COV-2, which is poorly understood at present.
4. To prevent or control the future spread of novel CoVs, global efforts are needed to construct a comprehensive global CoV database and timely warning system by collecting samples from humans and potential animal hosts (including bat species, which are both potentially natural hosts of CoVs and globally distributed).

## Data Availability Statement

The original contributions presented in the study are included in the article/Supplementary Material, further inquiries can be directed to the corresponding author/s.

## Author Contributions

ZZ was responsible for project planning, coordination, execution, and facilitation. XL and QW were responsible for modeling-based analysis. QW was responsible for data collection and phylogenetic analysis. ZZ, XL, and QW prepared the manuscript. All authors contributed to the article and approved the submitted version.

## Conflict of Interest

The authors declare that the research was conducted in the absence of any commercial or financial relationships that could be construed as a potential conflict of interest.

## Publisher’s Note

All claims expressed in this article are solely those of the authors and do not necessarily represent those of their affiliated organizations, or those of the publisher, the editors and the reviewers. Any product that may be evaluated in this article, or claim that may be made by its manufacturer, is not guaranteed or endorsed by the publisher.
