# Supplementary material for: Global Diversification and Distribution of Coronaviruses With Furin Cleavage Sites
Source: Front Microbiol. 2021 Oct 7;12:649314. doi: 10.3389/fmicb.2021.649314 (PMC8529245; doi:10.3389/fmicb.2021.649314)
Supplement: Supplementary file 3 [file Data_Sheet_1.docx]

***Supplementary Material***

# Supplementary Methods

***Obtaining the S protein sequences***

We retrieved the S protein sequences of 2297 coronaviruses from Genome Warehouse in the National Genomics Data Center (2020.2.20 update) (National Genomics Data Center and Partners, 2020). After aligning all the sequences using MAFFT (Katoh et al., 2002), those with poor alignment were removed.

***Prediction of furin cleavage sites from CoVs***

The filtered sequences were imported into ProP (v1.0) software (Duckert et al., 2004) that used to predict furin protease cleavage recognition site or furin cleavage site. Only those motifs consisting of 9 amino acids located in spike S1/S2 site with furin scores above 0.5 were regarded as confident furin cleavage sites.

***Phylogenetic analysis of the S proteins of CoVs with furin cleaveage sites.***

To trace evolutionary history of S proteins with furin cleavage sites, the maximum likelihood phylogenetic relationships of final S protein sequences were reconstructed using megaX (version 10.1.7) (Kumar et al., 2018) under the best-fit amino acid substitution model with 1000 bootstrap replications (Figure 1). The relationship of all furin cleavage site motif was shown using neighbor-joining (NJ) tree in megaX. All phylogenetic trees were visualized using iTOL (Letunic and Bork, 2019).

# Supplementary References

National Genomics Data Center, M., and Partners (2020). Database Resources of the National Genomics Data Center in 2020. *Nucleic Acids Res* 48(D1), D24-D33.

Katoh, K., Misawa, K., Kuma, K.-i., and Miyata, T. (2002). MAFFT: a novel method for rapid multiple sequence alignment based on fast Fourier transform. *Nucleic Acids Res* 30(14), 3059-3066.

Duckert, P., Brunak, S., and Blom, N. (2004). Prediction of proprotein convertase cleavage sites. *Protein Eng Des Sel* 17(1), 107-112.

Kumar, S., Stecher, G., Li, M., Knyaz, C., and Tamura, K. (2018). MEGA X: molecular evolutionary genetics analysis across computing platforms. *Mol Biol Evol* 35(6), 1547-1549.

Letunic, I., and Bork, P. (2019). Interactive Tree Of Life (iTOL) v4: recent updates and new developments. *Nucleic Acids Res* 47(W1), W256-W259.

# Supplementary Figures and Tables

**Supplementary Figure 1.** Phylogenetic tree of 9 amino-acid-length motifs representing 86 high-confident furin cleavage sites identified from the S protein sequences of 249 CoVs. The branches colored by virus genus (purple = Alpha-CoV; yellow = Beta-CoV; light blue = Gamma-CoV). Furin cleavage sites motifs from four CoVs known to infect humans (HCoV-OC43, HCoV-HKU1, MERS-CoV and SARS-CoV) and another human enteric coronavirus 4408(HECV-4408) first detected in Germany in 1988 were marked in red. And the first detected furin cleavage site motif in 1954 was marked in blue. **Linked to Supplementary Table 2**.

**Supplementary Table 1.** Information on SARS-CoV-2 and 248 other CoVs with high-confidence furin cleavage sites. Tree labels refers to labels in **Figure 1**, which shows the sites' phylogenetic relationships.

**Supplementary Table 2.** Eighty-six identified types of furin cleavage sites in CoVs. For a visualization of the sites' phylogenetic relationships see **Figure 1**.
