# Supplementary figures and images for: Global Diversification and Distribution of Coronaviruses With Furin Cleavage Sites
Source: Front Microbiol. 2021 Oct 7;12:649314. doi: 10.3389/fmicb.2021.649314 (PMC8529245; doi:10.3389/fmicb.2021.649314)

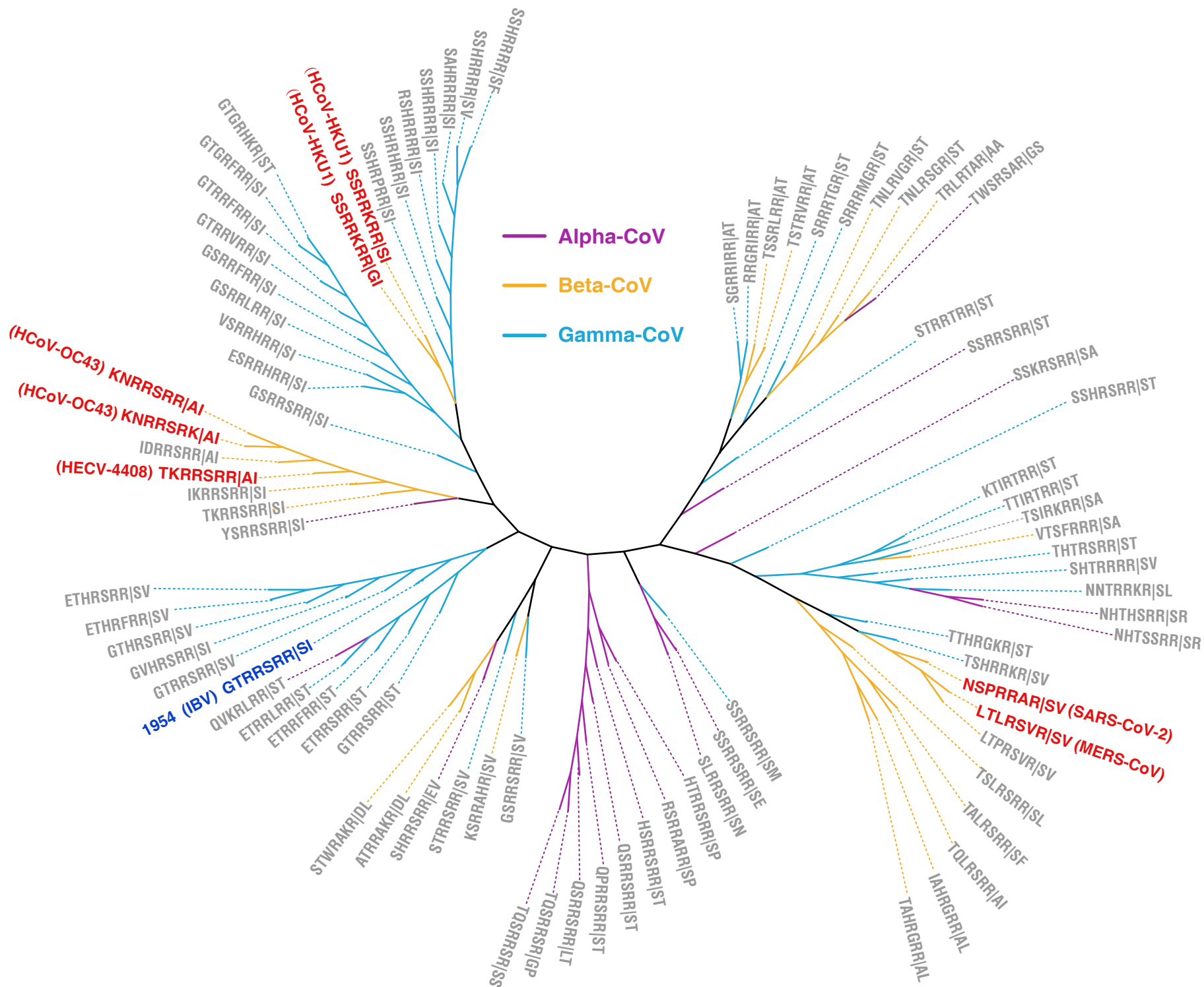

Supplement: Supplementary file 4 [file Image_1.pdf]
